# Influence of Chitosan Treatment on Surrogate Serum Markers of Cholesterol Metabolism in Obese Subjects

**DOI:** 10.3390/nu10010072

**Published:** 2018-01-11

**Authors:** Dieter Lütjohann, Milka Marinova, Karsten Wolter, Winfried Willinek, Norman Bitterlich, Martin Coenen, Christoph Coch, Frans Stellaard

**Affiliations:** 1 Institute for Clinical Chemistry and Clinical Pharmacology, University Clinics of Bonn, D-53127 Bonn, Germany; martin.coenen@ukb.uni-bonn.de (M.C.); ccoch@uni-bonn.de (C.C.); fstellaard@hotmail.com (F.S.); 2 Department of Radiology, University Clinics of Bonn, D-53127 Bonn, Germany; milka.marinova@ukb.uni-bonn.de (M.M.); karsten.wolter@ukb.uni-bonn.de (K.W.); w.willinek@bk-trier.de (W.W.); 3 Department of Radiology, Neuroradiology, Sonography and Nuclear Medicine, Krankenhaus der Barmherzigen Brüder Trier, D-54292 Trier, Germany; 4 Medizin & Service GmbH, Abt. Biostatistik, Boettcherstraße 10, D-09117 Chemnitz, Germany; bitterlich@medizinservice-sachsen.de

**Keywords:** cholesterol synthesis, bile acid synthesis, cholesterol absorption, lathosterol, plant sterols, oxysterols, lipoproteins, lipid lowering, phytosterols, placebo

## Abstract

Chitosan treatment results in significantly lower serum low density lipoprotein (LDL) cholesterol concentrations. To assess the working mechanisms of chitosan, we measured serum surrogate markers of cholesterol absorption (campesterol, sitosterol, cholestanol), synthesis (lathosterol, lanosterol, desmosterol), and degradation to bile acids (7α-hydroxy-cholesterol, 27-hydroxy-cholesterol), corrected for cholesterol concentration (R_sterols). Over 12 weeks, 116 obese subjects (Body Mass Index, BMI 31.7, range 28.1–38.9 kg/m^2^) were studied under chitosan (*n* = 61) and placebo treatments (*n* = 55). The participants were briefly educated regarding improvement of nutrition quality and energy expenditure. Daily chitosan intake was 3200 mg. Serum LDL cholesterol concentration decreased significantly more (*p* = 0.0252) under chitosan (−8.67 ± 18.18 mg/dL, 5.6%) than under placebo treatment (−1.00 ± 24.22 mg/dL, 0.9%). This reduction was not associated with the expected greater decreases in markers of cholesterol absorption under chitosan treatment. Also, increases in markers of cholesterol synthesis and bile acid synthesis under chitosan treatment were not any greater than under placebo treatment. In conclusion, a significant selective reduction of serum LDL cholesterol under chitosan treatment is neither associated with a reduction of serum surrogate markers of cholesterol absorption, nor with increases of markers for cholesterol and bile acid synthesis.

## 1. Introduction

Chitosan is a soluble fiber, which consists of polyglucosamine, produced by the deacetylation of chitin. The amino groups are protonated in an acidic environment. These hydrogen cations are able to bind to carboxylic compounds, like fatty acids and bile acids. This action is thought to reduce body weight and levels of serum lipids. However, reports on chitosan treatment in obese subjects have shown contradictory results regarding weight reduction and serum lipids [1,2,3]. Results appear to be dependent on many factors, such as chitosan product composition (percentage of deacetylation, content of vitamin C and tartaric acid) and dosage as well as duration of treatment, group size, degree of obesity of subjects and the presence of an accompanying weight reduction program. The intention for treatment with chitosan is the binding of fatty acids, cholesterol and bile acids in the stomach and intestine, followed by increased fecal excretion of fatty acids and cholesterol metabolites. In a number of human studies [4,5,6], serum total cholesterol was found to be not reduced, while in a Cochran analysis study [1], a meta-analysis found a small significant effect in favor of chitosan: −0.15 mmol/L (95% condidence interval, CI −0.23 to −0.07). Low density lipoprotein (LDL) cholesterol is commonly, but not always [4,6], reduced under chitosan treatment. Yet again, a small but significant effect in favor of chitosan was established (−0.16 mmol/L (95% CI −0.23 to −0.10)) in the same Cochran meta-analysis study [1]. However, this was not confirmed in a second meta-analysis study [7]. In animal studies, large increasing effects of chitosan on serum high density lipoprotein (HDL) cholesterol have been demonstrated [2], whereas in humans, the effect appears to be significant but marginal [1]: 0.03 mmol/L (95% CI 0.01 to 0.05). In many studies, no effects were found [4,8,9,10,11,12,13]. The mechanisms of action of chitosan have not been fully understood, to date. The major action is assumed to take place in the stomach, where protonation is favored by hydrogen production from the goblet cells. However, prior to the binding of free fatty acids, dietary triglycerides and phospholipids must be hydrolyzed by gastric lipase to free acids. Cholesterol and esterified cholesterol are not available in negatively ionized form. Under normal conditions, bile acids are not present in the stomach.

A further effect of chitosan is gel formation in the stomach [14,15,16]. Due to the high viscosity of the gastric content, gastric emptying is delayed and rapid satiety is established [17]. With a gradually increasing pH in the intestine and reduced ionic binding capacity of chitosan, the gel transforms into a precipitate. It is assumed that the gel and the precipitate trap lipids and bile acids, leading to increased fecal loss. However, in humans, increased fat excretion was not confirmed in all studies [18]. In humans, fecal cholesterol excretion was measured only by Maezaki et al. [19], who found no increase.

In mice, van Bennekum et al. [17] did not find increased fecal excretion, either of cholesterol, or of bile acids, under chitosan treatment. In addition, no reduction of fractional cholesterol absorption rate was found. In contrast, the authors found a decreased food intake under chitosan treatment.

In rats, chitosan led to increased fecal excretion of cholesterol and bile acids [16]. However, Fukada et al. [20] showed that chitosan affected bacterial bile acid metabolism in rats, while quantitative bile acid excretion remained unchanged.

In humans, the composition of fecal bile acids changed towards increased proportions of primary bile acids, while the total bile acid excretion rate remained unchanged [19]. Thus, the working mechanisms of chitosan are not clear to date, especially not in humans. Based on the expected increase in fecal excretion of cholesterol metabolites and bile acids, it may be hypothesized that the observed reduction of serum LDL cholesterol is accompanied by reduced cholesterol absorption and increased cholesterol and bile acid synthesis.

Therefore, in order to investigate the mechanisms of action for the hypocholesterolemic effect of chitosan in humans, we studied the effect of chitosan treatment on serum markers of cholesterol absorption (campesterol, sitosterol, cholestanol), cholesterol synthesis (lathosterol, lanosterol, desmosterol), and bile acid synthesis (7α-hydroxy-cholesterol, 27-hydroxy-cholesterol) in obese volunteers.

## 2. Materials and Methods

### 2.1. Study Design and Population

This study was part of a larger clinical trial, designed as a 12-week, single center, randomized, placebo-controlled, double-blind, and parallel group study. The protocol was carried out with methods according to the guidelines for Good Clinical Practice (GCP) and the Declaration of Helsinki. It was approved by the Ethics Commission of the University Clinics Bonn (111/13-AMG-ff). Written informed consent was obtained from all participants. The study was performed at the phase I study unit of Study Center Bonn (Christoph Coch), Institute of Clinical Chemistry and Clinical Pharmacology (Gunther Hartmann), University Clinics Bonn, Germany. The trial participants were recruited through advertisements in a daily newspaper, via wall posters presented at the wards as well as information posted on the University Clinics Bonn intranet. No dependent individuals were included in this trial. The main inclusion and exclusion criteria for this study were as follows: age 18–65 years, Body Mass Index (BMI) 28–36 kg/m^2^ at the time of presentation, waist circumference >88 cm (women) and >102 cm (men). The absence of relevant diseases, e.g., cardiovascular, hepatobiliary and gastrointestinal, previous or active malignant, neurological or psychiatric diseases or conditions after surgery, was documented. Excluded individuals included diabetes mellitus type 1 and 2 patients, subjects with actual or suspected alcohol or drug abuse, subjects with weight reduction >5 kg within the last five months and subjects known to be allergic to crustaceans. Women at child-bearing age had to present a negative pregnancy test and provide evidence of proper use of contraceptives or other factors preventing pregnancy to occur during the study. Subjects were not allowed to participate in other clinical trials.

After stratification according to gender, patients were assigned to the respective groups using appropriate block randomization. We started our study with 129 volunteers. However, we only had all data from 116 volunteers for the final evaluation of our intention-to-treat population. The chief investigator, other investigators, study staff, bioanalysts and participants were all blinded to the treatment allocation, in accordance with the double-blind design.

Participants in the chitosan group (*n* = 61) received eight chitosan-containing tablets (Biopolymer3200, Certmedica International GmbH, Aschaffenburg, Germany), which were taken twice a day, as four tablets, with the main meal. Biopolymer 3200 tablets consist of (β-1,4-polymer of d-glucosamine and *N*-acetyl-d-glucosamine containing >80% chitosan, 5–10% vitamin C, 1–5% tartaric acid and 5–10% water.

The 55 participants in the placebo group received eight placebo tablets to be divided over two meals, which contained 122.50 mg microcrystalline cellulose, 372.50 mg calcium hydrogen phosphate, 5.00 mg magnesium stearate, 0.750 mg iron oxide yellow, 0.375 mg iron oxide, brown, 0.375 mg iron oxide black per tablet. During the study (nine follow-up visits), the remaining tablets were counted to check for compliance.

All subjects were advised to reduce their fat intake to 60–80 g fat per day and their energy intake by 500 kcal per day. During the first ten weeks of the study program, the participants participated in an eight-session nutrition information course, presented by professional dieticians. The intention of the course was to familiarize the subjects with their personal dietary habits and relate these to recommended standards for a healthy lifestyle. The program consisted of eight PowerPoint presentations explaining causes of becoming overweight and risks that being overweight has on the development of chronic diseases. Also, aspects of diet composition, energy intake and expenditure were presented.

The guidelines of the German Nutrition Society were followed [21]. At each session, food intake and body weight were recorded. The subjects were provided with a take-home version of the PowerPoint presentations and a DVD with exercise recommendations. Adaption to the recommended diet was not monitored. All subjects continued the study program independent of personal follow-up to improve or not improve their lifestyle during the study program.

### 2.2. Blood Sampling and Sterol Analysis

Fasting blood samples were collected with S-Monovette^®^ (7.5 mL, serum gel with clotting activator; Sarstedt AG & Co., Nümbrecht, Germany), before and after, 12 weeks of treatment. After centrifugation serum concentrations of total, HDL and LDL cholesterol were determined enzymatically in the central laboratory at the Institute of Clinical Chemistry and Clinical Pharmacology of the University Clinics of Bonn, accredited according to DIN EN ISO 15189:2014. Lipoprotein analyses are subject to internal quality control within the central laboratory and external quality assessment during successful participation in accredited ring trials evaluated by the German Reference Institute for Bioanalytics (RfB, Bonn, Germany), accredited according to DIN EN ISO/IEC 17043:2010.

All samples were analyzed with Siemens Dimension Vista^®^ 1500 Intelligent Lab System (Siemens Healthcare GmbH, Erlangen, Germany) using Dimenson Vista Flex™ reagent cartridges (Siemens Healthcare GmbH, Erlangen, Germany). All procedures were performed according to the instruction leaflets for the different lipoprotein cholesterol Flex^®^ reagent cartridges. After deacylation, free total cholesterol is oxidized in a reaction catalyzed by cholesterol oxidase to form cholest-4-ene-3-one and hydrogen peroxide. The latter oxidizes *N*,*N*-diethylaniline-HCL/4-aminoantipyrine to produce a chromophore that absorbs at 540 nm. This absorbance is directly proportional to the total cholesterol concentration (K1027, Siemens Flex^®^ reagent cartridge; Siemens Healthcare GmbH, Erlangen, Germany). The HDL assay (K3048A, Siemens Flex^®^ reagent cartridge; Siemens Healthcare GmbH, Erlangen, Germany) uses a two-reagent format. Dextran sulfate, in the presence of magnesium sulfate, complexes with chylomicrons, very low density lipoproteins (VLDL) and LDL. The residual HDL cholesterol esters are deacylated by polyethylene glycol-modified cholesterol esterase and the free HDL cholesterol is oxidized to ∆4-cholestenone and hydrogen peroxide. The latter reacts with 4-aminoantipyrine and *N*-(2-hydroxy-3-sulfopropyl)-3.5-dimethoxyaniline in the presence of peroxidase to a colored dye that is measured using a bichromatic technique. The LDL cholesterol assay (K3131, Siemens Flex^®^ reagent cartridge; Siemens Healthcare GmbH, Erlangen, Germany) is a homogenous method for directly measuring serum LDL cholesterol levels. The method is in a two-reagent format and depends on the properties of a detergent which solubilizes only non-LDL particles in the first step. Detergent 2 solubilizes the remaining LDL particles. The soluble LDL cholesterol is then oxidized by the actions of cholesterol esterase and cholesterol oxidase, forming cholestenone and hydrogen peroxide. The enzymatic actions of peroxidase produce color in the presence of *N*,*N*-bis(4-sulfobutyl)-m-toluidine, disodium salt and 4-aminoantipyrine that is measured using a bichromatic endpoint technique.

The serum concentrations of the surrogate markers of cholesterol absorption (campesterol, sitosterol, cholestanol), cholesterol synthesis (lathosterol, lanosterol, desmosterol) and bile acid synthesis (7α-hydroxy-cholesterol, 27-hydroxy-cholesterol) were measured with gas chromatography-mass spectrometry-selected ion monitoring [22,23]. The trimethylsilyethers of the sterols and oxysterols were separated on a DB-XLB (30 m length × 0.25 mm internal diameter, 0.25 μm film) column (Agilent Technologies, Waldbronn, Germany) using the 6890N Network GC system (Agilent Technologies, Waldbronn, Germany). Epicoprostanol (Steraloids, Newport, RI, USA) was used as an internal standard, to quantify the non-cholesterol sterols, and deuterium labeled oxysterols (Medical Isotopes, Pelham, NH, USA) were used for the isotope dilution-mass selective detection and quantification (MSD) of the bile acid precursors on a 5973 Network MSD (Agilent Technologies, Waldbronn, Germany). In order to correct these markers for total cholesterol from the same sample, we measured total cholesterol by gas chromatography-flame ionization detection on an HP 6890 GC system (Hewlett Packard, Waldbronn, Germany), equipped with a DB-XLB (30 m length × 0.25 mm internal diameter, 0.25 μm film) column (Agilent Technologies, Waldbronn, Germany) using 5a-cholestane (Steraloids, Newport, RI, USA) as internal standard [24]. These ratios, indicated as R_sterols or R_oxysterols, were used as markers of cholesterol absorption, synthesis and catabolism (=bile acid synthesis). Measurement of sterols and oxysterols were evaluated according to good laboratory practice and the measurements were supervised by an internal quality control system.

### 2.3. Statistics

Data are given as mean ± SD the changes initiated by chitosan and placebo treatments were tested against baseline, using the two-tailed Wilcoxon test. The changes under chitosan and placebo treatments were compared using the two-tailed Mann-Whitney U test. This was done for the total group as well as for the groups of subjects experiencing an increase or decrease. The frequencies of treatment responses were tested with Fisher’s exact test. The correlation between the change of parameter and the baseline parameter value before treatment was analyzed by Spearman’s correlation. The slopes and intercepts under chitosan treatment were compared with values under placebo treatment using linear regression analysis. Statistical comparisons, correlations and linear regression analyses were made with Graphpad Prism 5 (San Diego, CA, USA).

### 2.4. Interpretation of Results

The results must show whether chitosan treatment affects body weight, serum cholesterol concentrations, cholesterol absorption, synthesis and catabolism in a mode that is health-promoting and stronger than placebo treatment. The following evaluation approaches were applied:

1. Comparison of chitosan-induced changes with placebo-induced changes, i.e., the traditional approach in a placebo-controlled study.
2. Monitoring of the relative number of subjects who experienced an increase or decrease in the parameter. In addition to the mean chitosan-induced effect, the number of subjects achieving this change must be larger under chitosan treatment than under placebo treatment.
3. Assessment of the dependency of the observed change on the baseline value before treatment. A dependency under chitosan treatment indicates that an individual treatment effect can be predicted by the baseline value. The target patient group for treatment can be defined. Different dependencies under chitosan and placebo treatments may indicate different mechanisms leading to the observed changes.

## 3. Results

### 3.1. Comparison of the Baseline Data of the Chitosan Treatment and the Placebo Treatment Groups

For all studied parameters, no statistically significant differences were found between the chitosan group before treatment and the placebo group before treatment (Table 1).

### 3.2. Weight and Body Mass Index (BMI)

**Figure 1:**
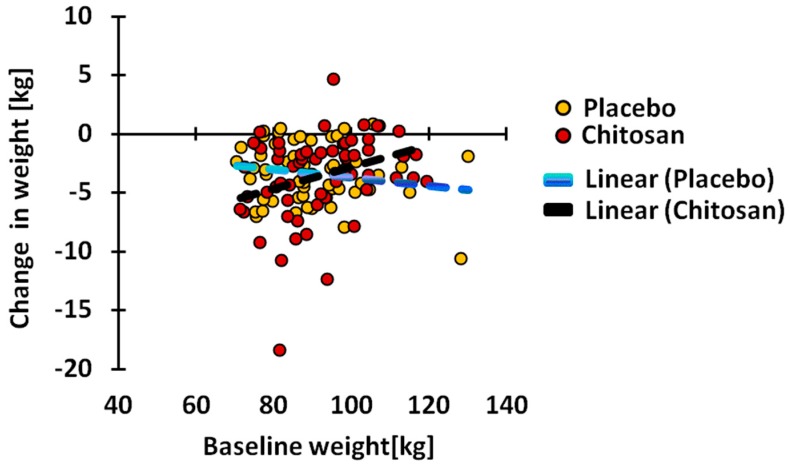
Dependence of the change in weight on baseline weight values under chitosan and placebo treatments. Under chitosan treatment, the change was significantly (*p* = 0.0083) and positively associated with baseline values.

The reduction in weight (Table 2) and BMI (Table 3) was statistically significant with the placebo (*p <* 0.0001) and chitosan treatment (*p <* 0.0001). The changes found in the placebo group and in the chitosan group were not statistically different from each other. During placebo treatment, 90.9% of subjects, and during chitosan treatment, 88.5% of the subjects, experienced weight reduction, while, 81.8% and 80.3%, respectively, experienced a reduction in BMI. The degree of weight reduction under chitosan treatment was highly and positively dependent (Figure 1) on the baseline value before treatment (Spearman R = 0.3349, *p* = 0.0083). This was not the case in placebo-treated subjects. BMI reduction was not associated with the baseline value, either under chitosan or under placebo treatment.

### 3.3. Serum Total Cholesterol

While serum total cholesterol (Table 4) did not decrease under placebo treatment (−5.13 ± 24.79 mg/dL, NS) it decreased significantly under chitosan treatment (−12.51 ± 28.22 mg/dL, *p* = 0.0007). Both changes did not significantly differ from each other. The number of subjects undergoing a decrease was also not different: 63.0% under placebo treatment and 67.2% during chitosan treatment. Both treatments led to a significant reduction that was negatively associated with the baseline value. Neither slopes, nor intercepts, were significantly different.

### 3.4. Serum Low Density Lipoprotein (LDL) Cholesterol

**Figure 2:**
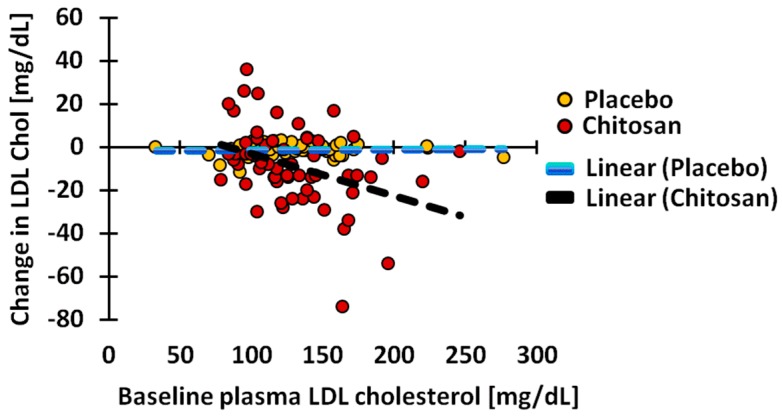
Dependence of change of serum low density lipoprotein (LDL) cholesterol concentration on baseline values under chitosan and placebo treatment. Under chitosan treatment, the change is significantly (*p* = 0.0014) and negatively associated with the baseline value.

Serum LDL cholesterol (Table 5) decreased significantly under chitosan (−8.67 ±18.18 mg/dL, *p* = 0.0003), but not under placebo treatment (−1.00 ± 24.22 mg/dL, *p* = 0.5613). The reduction induced by chitosan was significantly larger than the reduction induced by placebo (*p* = 0.0252). During placebo treatment, the LDL cholesterol concentration decreased in 48.2% of the subjects, while during chitosan treatment, the value was reduced in 73.8% (*p* = 0.0076). Interestingly, the mean reduction in the subjects undergoing a reduction, and the mean increase in subjects showing an increase, were both significantly higher in the chitosan-treated group (both *p <* 0.0001) than in the placebo group. Only in chitosan-treated subjects was the change highly significantly (*p* = 0.0014) and negatively associated with baseline value (Figure 2).

### 3.5. Serum High Density Lipoprotein (HDL) Cholesterol

Serum HDL cholesterol did not significantly change under placebo or chitosan treatments (Table 6). The observed changes in both treatment groups did not differ from each other. Also, the number of subjects experiencing a decrease or increase was similar.

### 3.6. Cholesterol Absorption Markers

Due to significant decreases in serum total cholesterol under placebo as well as chitosan treatment, only the marker concentrations corrected for the cholesterol concentrations of R_campesterol, R_sitosterol, and R_cholestanol were considered. The changes of the cholesterol absorption marker sterols (Table 7, Table 8 and Table 9) during both treatments were not significant from zero, except for a reduction in R_cholestanol (Table 9) under placebo treatment. Also, changes found under chitosan treatment did not differ from those found under placebo treatment. For all three marker compounds, the changes were significantly and negatively associated with baseline values in both groups. However, neither slopes, nor intercepts, differed between treatment groups.

### 3.7. Cholesterol Synthesis Markers

Due to the significant decreases in serum total cholesterol under placebo as well as chitosan treatment, only the marker concentrations corrected for the cholesterol concentrations of R_lathosterol, R_lanosterol, and R_desmosterol were considered (Table 10, Table 11 and Table 12). R_lathosterol significantly decreased only under chitosan treatment (*p* = 0.0334). The difference between chitosan and placebo treatments was not significant (*p* = 0.0759). R_lathosterol decreased in 49.1% of the subjects under placebo treatment and in 59.0% of subjects under chitosan treatment (NS). R_lanosterol and R-desmosterol did not change significantly and no differences were found comparing both treatment changes. During both treatments, a negative association between change and baseline value was observed for all three markers, while slopes did not differ. In contrast to R_lathosterol and R_demosterol, increases in the placebo and the chitosan group were found for R_lanosterol. In subjects who experienced a decrease in R_lanosterol, the decrease was significantly less under chitosan treatment (*p* = 0.0324). However, relatively more chitosan-treated subjects experienced a decrease: 55.7% vs. 38.2% in placebo-treated subjects. This difference did not reach significance (*p* = 0.0654).

### 3.8. Bile Acid Synthesis Markers

R_7α-hydroxy-cholesterol was only significantly reduced under chitosan treatment (*p* = 0.0196) (Table 13). The changes induced by both treatments did not differ significantly from each other. During placebo treatment, R_7α-hydroxy-cholesterol was reduced in 67.3% of subjects, while during chitosan treatment, this was the case in 60.7% of the subjects (NS). No significant changes were seen regarding R_27-hydroxycholesterol in both groups (Table 14). The changes induced by the two treatments did not differ significantly from each other. The relationships between changes and baseline values did not differ between placebo and chitosan treatments, either for R_7α-hydroxy-cholesterol, or for R_27-hydroxy-cholesterol.

## 4. Discussion

The present study describes changes found in serum sterols in 61 highly overweight and obese subjects after chitosan treatment. It is a placebo-controlled study with a placebo group consisting of 55 subjects. Body weight, BMI, serum cholesterol concentrations and cholesterol-corrected sterol concentrations did not differ between both treatment groups before treatment.

### 4.1. Body Weight

A highly significant decrease in weight and BMI was found under both treatments. However, these decreases were not more pronounced under chitosan treatment when compared to placebo treatment. The percentage of subjects undergoing a decrease was also similar in both treatment groups. These data confirm human data obtained in previous studies [5,12,25]. Interestingly, under chitosan, but not placebo treatment, the weight change was highly significantly and positively associated with baseline weight values, indicating that the highest reduction is obtained at the lowest weight. Possibly, the selection of strongly overweight and obese subjects was not the best choice for demonstrating a weight reduction effect of chitosan treatment. In fact, chitosan treatment may be most efficient in weight gain prevention therapy in subjects who are overweight. The correlation data also suggest that the weight reductions that occurred due to placebo and chitosan treatments were established by different mechanisms.

### 4.2. Serum Cholesterol Concentrations

Serum total cholesterol decreased significantly, only under chitosan treatment. While this decrease did not significantly differ from the one observed with placebo treatment, it was much more pronounced (*p* = 0.0007) when compared to the placebo treatment (*p* = 0.0553). Therefore, a partial chitosan-dependent effect could be assumed. However, the percentage of subjects undergoing total cholesterol reduction was only slightly higher in the chitosan treatment group. The correlation data did not indicate any trend to indicate that different mechanisms could explain the concentration reduction under placebo and under chitosan treatments. The decrease in LDL cholesterol was greater under chitosan treatment than under placebo treatment (*p* = 0.0252). Compared to the baseline situation, only chitosan induced a significant decrease (*p <* 0.0003) in this parameter. Also, the percentage of subjects experiencing a decrease of LDL cholesterol was significantly higher in subjects from the chitosan treatment group (73.8%) than in subjects from the placebo treatment group (48.2%). Thus, according to all three criteria, a clear chitosan-induced 5.6% reduction of LDL cholesterol was achieved, compared to a reduction of 0.9% under placebo treatment. Assuming that the reduction of LDL cholesterol is solely due to trapping of dietary cholesterol in the stomach and intestine, it is of interest to relate this number to the 13% LDL cholesterol reduction found in vegan subjects, who, compared to omnivores, ingest 90% less cholesterol through their diet [26]. In lacto-vegetarians, cholesterol intake is 44% lower than in omnivores, but lower serum LDL cholesterol values are not found [26]. Based on these findings, a trapping efficiency of 60–70% of dietary cholesterol is predicted under chitosan treatment.

### 4.3. Surrogate Markers of Cholesterol Absorption

The major serum markers of cholesterol absorption are cholestanol and the plant sterols—campesterol and sitosterol. Plant sterols are known to undergo similar changes in absorption to cholesterol. Under chitosan treatment, only the campesterol concentration showed selectively lowered values. However, serum cholesterol decreased significantly under both treatments. After correcting the plant sterol concentrations for the cholesterol concentrations, no significant differences remained. Also, no differences were found between changes due to chitosan and placebo. In a recent publication [27], we showed that the plant sterol/cholesterol ratio is a good and sensitive reflection of the fractional cholesterol absorption rate measured with stable isotope tracers. Comparing vegan subjects with omnivores [26] did lead to a slightly, but significantly, lower fractional cholesterol absorption rate in vegans (42% vs. 50%) and a greatly lowered (90%) dietary cholesterol intake, but not to a change in R_campesterol, R_sitosterol or R_cholestanol. This may be due to a potentially high intake of plant sterols in vegans. Ezetimibe treatment leads to a more than 50% reduction in the fractional cholesterol absorption and significantly reduced levels of R_campesterol and R_sitosterol, but not R_cholestanol [27]. Importantly, ezetimibe also affects the absorption of biliary cholesterol, which amounts to 2–3 times more than dietary cholesterol, whereas a vegan diet and possibly also chitosan treatment affects only dietary cholesterol. In view of the available data, these results suggest that, unlike ezetimibe, chitosan treatment does not significantly affect cholesterol absorption.

### 4.4. Surrogate Markers of Cholesterol Synthesis

Three markers of cholesterol synthesis were measured in serum: lathosterol, desmosterol and lanosterol. Changes observed for desmosterol and lanosterol disappeared after correction for the cholesterol concentration. The ratio of R_lathosterol decreased under chitosan treatment but not under placebo treatment. The decrease observed for chitosan treatment was not significantly different from the decrease observed for placebo treatment (*p* = 0.0759). Of the chitosan-treated subjects, 59% had lower R_lathosterol, which was not significantly higher than the 49% of subjects found in the placebo group. The results can be interpreted as an indication of a small 3% decrease in cholesterol synthesis under chitosan treatment. At any rate, the data do not indicate increased synthesis, as hypothesized. The results may be compared with data in lacto-vegetarians and vegans, as recently described and measured with stable isotope techniques [26]. Lacto-vegetarians had a 22% higher cholesterol synthesis than omnivores without a reduction in LDL cholesterol, and vegans, a 35% higher synthesis and a 13% lower LDL cholesterol. However, these diet-induced differences in cholesterol synthesis did not lead to modifications in the R_desmosterol and R_lathosterol ratios. As described before [27,28], the surrogate markers—R_lathosterol and R_desmosterol—for cholesterol synthesis are not sensitive enough to detect relatively small changes in whole body synthesis during cholesterol lowering therapy, as they primarily reflect hepatic synthesis. Lowering daily intake of cholesterol or fractional absorption of cholesterol may lead to a preferentially enhanced synthesis in intestinal cells. These data do not indicate increased cholesterol synthesis, as hypothesized.

### 4.5. Surrogate Markers of Bile Acid Synthesis

7α- and 27-hydroxy-cholesterol are markers for bile acid synthesis, with 7α-hydroxy-cholesterol representing the major route of bile acid synthesis. Under chitosan treatment, the ratio of R_7α-hydroxy-cholesterol was significantly reduced (*p* = 0.0196), but not significantly more so than under placebo treatment. R_27-hydroxy-cholesterol did not change significantly during both treatments and both changes were not different. The percentages of subjects undergoing R_7α- or R_27-hydroxy-cholesterol reduction or increase did not differ under both treatments. The associations between change and baseline value were significant for 7α-hydroxy-cholesterol and 27-hydroxy-cholesterol under placebo as well as chitosan treatment. However, neither slopes, nor intercepts, differed under both treatments. Therefore, our data do not support an independent chitosan effect on bile acid synthesis.

### 4.6. Placebo Effects vs. Chitosan Effects

R_cholestanol decreased significantly under placebo treatment. Other sterols (serum total cholesterol, LDL cholesterol, R_lathosterol and R_7α-hydroxycholesterol), were significantly reduced, only under chitosan treatment, suggesting an independent chitosan effect. However, the changes during both treatments were not significantly different, except for LDL cholesterol. Therefore, the reduction of serum LDL cholesterol under chitosan treatment was the only confirmed independent effect. Body weight and BMI were also significantly reduced during placebo treatment. These reductions can be explained by the fact that the participants had been advised on how to improve the quality of their food intake and on energy expenditure. However, they could eat as usual and, more importantly, dietary compliance was not monitored. Interestingly, the reductions in weight and BMI did not differ between placebo and chitosan-treated subjects. Significant changes compared to baseline were observed in both groups (*p <* 0.0001). If the nutritional information provided to the subjects is to be considered as the cause of the weight reduction, the mechanism of action should be the same for both treatment groups. However, the significant positive association between the change in body weight and baseline value under chitosan treatment suggests a selective mechanism of action. The question remains as to whether the placebo tablet composition may have led to effects. The 55 subjects receiving placebo ingested eight times 122.50 mg or 980 mg microcrystalline cellulose and eight times 372.50 mg or 2980 mg calcium hydrogenphosphate per day. Cellulose is a solid non-soluble fiber, with a low, but potential, capacity to bind sterols. Cellulose, non-digestible for humans, is fuel for the colonic microbiota, and one product of their fermentation are the short-chain fatty acids influencing health, blood lipid profiles and reducing body weight [29]. Calcium hydrogenphosphate is a proton donor applied in baking powder. The potential effect of a daily dosage of 3 g cannot be simply predicted.

The dose of chitosan applied in this study was four times the dose used in another study with the same chitosan product, but in combination with a high protein formula replacement of a meal once a day [12]. The placebo group also consumed the meal replacement. The same placebo tablet was used as in the present study, but at a four times lower dose. In this study, serum total cholesterol and LDL cholesterol significantly decreased only in the chitosan treatment group. In both cases, the changes introduced by chitosan were significantly larger than by placebo.

The results of the present study do not provide an explanation for the reduced serum LDL cholesterol concentration under chitosan treatment. The hypothesis that chitosan treatment creates a reduced absorption of dietary cholesterol, partly compensated by an increased cholesterol synthesis rate, could not be proven when applying the surrogate marker technology. The question remains as to whether the applied experimental protocol and the measurement of surrogate markers for cholesterol absorption and synthesis are sufficiently appropriate to test this hypothesis. From previous studies, it could have been predicted that reductions in serum total cholesterol and LDL cholesterol would be small, in the order of a few percent. The choice of a placebo-controlled study implies the difficulty of adequately differentiating between a placebo effect and a selective chitosan effect. The difficulty is compounded when the differences are small. Discussion on the validity of surrogate markers for cholesterol absorption and synthesis under cholesterol lowering therapies is ongoing [27,28]. In particular, the sensitivity of cholesterol synthesis markers may be considered too low to detect small changes. Furthermore, these markers are considered to represent hepatic cholesterol synthesis. A computer-randomized, double-blind, placebo-controlled, four-period, balanced, crossover study should be initiated, combined with appropriate measurement of daily cholesterol intake and fecal excretion of neutral and acidic sterols as well as plant sterols. A continuous stable isotope feeding method, to accurately determine the fractional cholesterol absorption and cholesterol balance procedure, to measure cholesterol synthesis should be applied. This approach will give maximum information on independent effects of chitosan, in particular when participants are fed at the metabolic ward with a strictly controlled diet. Using the same approach, various dependencies, such as the chosen chitosan product (composition, dose, % deacetylation, viscosity index), body weight of studied subjects and experimental conditions (caloric restriction, altered diet composition, altered energy expenditure, normo vs. hypercholesterolemic state) should be investigated. Based on these findings, the optimal formula and the optimal target patient group for treatment can be identified.

Recently, interesting alternative modes of action of chitosan have been presented [2] that may affect cholesterol metabolism, independently of absorption and synthesis. Some of the mechanisms may be based on general characteristics of fibers: delay of gastric emptying, increased satiety, reduction in appetite, modulation of incretin secretion. Apparently, chitosan treatment can lead to delayed gastric emptying through the highly viscous gel formation and to increased satiety. The latter may lead to decreased food intake as was shown in mice [17]. Most human studies on chitosan effects deal with effects on body weight, BMI and waist circumference and/or serum lipid concentrations. Food intake is not generally assessed under treatment. Maezaki et al. [19] showed data from a two-week period, treating eight normal weight subjects, with chitosan incorporated in biscuits, which indicated that cholesterol intake decreased from 340 to 276 mg/day, albeit not statistically significantly so. The question remains as to what might have happened after a longer chitosan treatment duration. Chitosan has also been shown to act antibacterially [30,31] and to affect colonic fermentation in rats [32], including short chain fatty acid production, which may reduce cholesterol synthesis via propionic acid. Chitosan also acts as an antioxidant [33,34].

## 5. Conclusions

A 12-week treatment of highly overweight and obese subjects with 3 g/day chitosan resulted in significantly lowered serum LDL cholesterol, but it did not alter surrogate serum markers of cholesterol absorption, synthesis and catabolism. A small reduction of dietary cholesterol absorption cannot be excluded.

## Figures and Tables

**Table 1:** Comparison of baseline data (mean ± SD) of the chitosan group and placebo group.

	Placebo	Chitosan	*p*-Value Chitosan vs. Placebo ^#^
Weight (kg)	93.3 ± 13.8	95.7 ± 11.6	0.1594
BMI (kg/m^2^)	31.6 ± 2.3	31.8 ± 2.3	0.6864
Serum total cholesterol (mg/dL)	216 ± 49.7	209 ± 42.5	0.3430
Serum LDL cholesterol (mg/dL)	131 ± 39.8	129 ± 35.3	0.7213
Serum HDL cholesterol (mg/dL)	54.7 ± 16.8	53.5 ± 13.9	0.7525
R_campesterol (μg/mg)	21.6 ± 5.73	22.3 ± 5.75	0.3515
R_sitosterol (μg/mg)	1.24 ± 0.56	1.25 ± 0.47	0.8552
R_cholestanol (μg/mg)	1.08 ± 0.29	1.14 ± 0.34	0.2629
R_lathosterol (μg/mg)	1.66 ± 0.66	1.62 ± 0.53	0.7758
R_lanosterol (ng/mg)	141 ± 40.1	132 ± 30.4	0.3360
R_desmosterol (μg/mg)	0.76 ± 0.35	0.70 ± 0.19	0.7090
R_7αOH-cholesterol (μg/mg)	21.5 ± 46	22.2 ± 13.2	0.8466
R_27OH-cholesterol (μg/mg)	74.7 ± 16.7	78.9 ± 19.8	0.2367

^#^ Data are expressed as *p*-values obtained with the Mann-Whitney test. BMI, Body Mass Index; LDL, low density lipoprotein.

**Table 2:** Comparison of changes in body weight induced by chitosan and placebo treatments.

	Placebo ^∆^	Chitosan ^∆^	*p*-Value Chitosan vs. Placebo
All subjects (kg)	−3.35 ± 2.51 ***	−3.51 ± 3.64 ***	0.7234 ^#^
% subjects decrease	90.9	88.5	0.7660 ^#^
% subjects increase	9.1	11.5	
Change vs. baseline			
Spearman R	−0.0918	0.3349	
Spearman *p*-value	0.5051	0.0083	
Difference in slope (*p*-value )	0.1796 ^$^	0.0157 ^$^	0.0060 ^&^

^∆^ Wilcoxon *p*-value, expressing the significance of the change compared to zero, *** *p <* 0.001, ^#^ data are expressed as *p*-values using the Mann–Whitney test, ^$^ value expresses whether slope is different from zero, ^&^ value expresses whether slopes under chitosan and placebo treatments are different.

**Table 3:** Comparison of changes in BMI resulting from chitosan and placebo treatments.

	Placebo ^∆^	Chitosan ^∆^	*p*-Value Chitosan vs. Placebo
All subjects (kg/m^2^)	−1.08 ± 0.89 ***	−0.95 ± 1.73 ***	0.539 ^#^
% subjects decrease	81.8	80.3	1.000 ^#^
% subjects increase	18.2	19.7	
Change vs. baseline			
Spearman R	−0.2397	−0.1505	
Spearman *p*-value	0.0780	0.2470	
Difference in slope ( *p*-value)	0.0781 ^$^	0.1703 ^$^	0.7124 ^&^

^∆^ Wilcoxon *p*-value expressing the significance of the change compared to zero, *** *p <* 0.001, ^#^ data are expressed as *p*-values using the Mann–Whitney test, ^$^ value expresses whether slope is different from zero, ^&^ value expresses whether slopes under chitosan and placebo treatments are different.

**Table 4:** Comparison of changes in serum total cholesterol induced by chitosan and placebo treatments.

	Placebo ^∆^	Chitosan ^∆^	*p*-Value Chitosan vs. Placebo
All subjects (mg/dL)	−5.13 ± 24.79	−12.51 ± 28.22 ***	0.3336 ^#^
% subjects decrease	63.0	67.2	0.6968 ^#^
% subjects increase	37.0	32.8	
Change vs. baseline			
Spearman R	−0.4792	−0.3587	
Spearman *p*-value	0.0002	0.0045	0.9399
Difference in slope (*p*-value)	0.0002 ^$^	0.0066 ^$^	0.0553 ^&^

^∆^ Wilcoxon *p*-value expressing the significance of the change compared to zero, *** *p <* 0.001, ^#^ data are expressed as *p*-values using the Mann–Whitney test, ^$^ value expresses whether slope is different from zero, ^&^ value expresses whether slopes under chitosan and placebo treatments are different.

**Table 5:** Comparison of changes in serum low density lipoprotein (LDL) cholesterol induced by chitosan and placebo treatments.

	Placebo ^∆^	Chitosan ^∆^	*p*-Value Chitosan vs. Placebo
All subjects (mg/dL)	−1.00 ± 24.22	−8.67 ± 18.18 ***	0.0252 ^#^
% subjects decrease	48.2	73.8	0.0076 ^#^
% subjects increase	51.8	26.2	
Change vs. baseline			
Spearman R	0.03768	−0.3995	
Spearman *p*-value	0.7868	0.0014	
Difference in slope (*p*-value)	0.7797 ^$^	0.0024 ^$^	0.0019 ^&^

^∆^ Wilcoxon *p*-value expressing the significance of the change compared to zero, *** *p <* 0.001, ^#^ data are expressed as *p*-values using the Mann–Whitney test, ^$^ value expresses whether slope is different from zero, ^&^ value expresses whether slopes under chitosan and placebo treatments are different.

**Table 6:** Comparison of changes in serum high density lipoprotein (HDL) cholesterol induced by chitosan and placebo treatments.

	Placebo ^∆^	Chitosan ^∆^	*p*-Value Chitosan vs. Placebo
All subjects (mg/dL)	−1.06 ± 6.81	−1.15 ± 7.65	0.8701 ^#^
% subjects decrease	55.6	52.5	0.8516 ^#^
% subjects increase	44.4	47.5	
Change vs. baseline			
Spearman R	−0.5054	−0.1491	
Spearman *p*-value	<0.0001	0.2513	
Difference in slope (*p*-value)	0.0004 ^$^	0.1810 ^$^	0.9648 ^&^

^∆^ Wilcoxon *p*-value expressing the significance of the change compared to zero, ^#^ data are expressed as *p*-values using the Mann–Whitney test, ^$^ value expresses whether slope is different from zero, ^&^ value expresses whether slopes under chitosan and placebo treatments are different.

**Table 7:** Comparison of changes in serum R_campesterol induced by chitosan and placebo treatments.

	Placebo ^∆^	Chitosan ^∆^	*p*-Value Chitosan vs. Placebo
All subjects (μg/mg)	−0.04 ± 0.51	−0.12 ± 0.50	0.3333 ^#^
% subjects decrease	50.9	55.7	0.7097 ^#^
% subjects increase	49.1	44.3	
Change vs. baseline			
Spearman R	−0.4901	−0.4040	
Spearman *p*-value	0.0001	0.0012	
Difference in slope (*p*-value)	<0.0001 ^$^	0.0084 ^$^	0.6112 ^&^

^∆^ Wilcoxon *p*-value expressing the significance of the change compared to zero, ^#^ data are expressed as *p*-values using the Mann–Whitney test, ^$^ value expresses whether slope is different from zero, ^&^ value expresses whether slopes under chitosan and placebo treatments are different.

**Table 8:** Comparison of changes in serum R_sitosterol induced by chitosan and placebo treatments.

	Placebo ^∆^	Chitosan ^∆^	*p*-Value Chitosan vs. Placebo
All subjects (μg/mg)	−0.06 ± 0.27	−0.06 ± 0.36	0.9471 ^#^
% subjects decrease	52.7	54.1	1.0000 ^#^
% subjects increase	47.3	15.9	
Change vs. baseline			
Spearman R	−0.4224	−0.3557	
Spearman *p*-value	0.0013	0.0049	
Difference in slope (*p*-value)	<0.0001 ^$^	0.0027 ^$^	0.9860 ^&^

^∆^ Wilcoxon *p*-value expressing the significance of the change compared to zero, ^#^ data are expressed as *p*-values using the Mann–Whitney test, ^$^ value expresses whether slope is different from zero, ^&^ value expresses whether slopes under Chitosan and placebo treatments are different.

**Table 9:** Comparison of changes in serum R_cholestanol induced by chitosan and placebo treatments.

	Placebo ^∆^	Chitosan ^∆^	*p*-Value Chitosan vs. Placebo
All subjects (μg/mg)	0.07 ± 0.26 *	0.02 ± 0.24	0.255 ^#^
% subjects decrease	30.9	42.6	0.2485 ^#^
% subjects increase	69.1	57.4	
Change vs. baseline			
Spearman R	−0.4346	−0.5283	
Spearman *p*-value	0.0009	*p <* 0.0001	
Difference in slope (*p*-value)	0.0009 ^$^	<0.0001 ^$^	0.9082 ^&^

^∆^ Wilcoxon *p*-value expressing the significance of the change compared to zero, * *p <* 0.05, ^#^ data are expressed as *p*-values using the Mann–Whitney test, ^$^ value expresses whether slope is different from zero, ^&^ value expresses whether slopes under chitosan and placebo treatments are different.

**Table 10:** Comparison of changes in serum total R_lathosterol induced by chitosan and placebo treatments.

	Placebo ^∆^	Chitosan ^∆^	*p*-Value Chitosan vs. Placebo
All subjects (μg/mg)	0.01 ± 0.40	−0.11 ± 0.37 *	0.0759 ^#^
% subjects decrease	49.1	59.0	0.3513 ^#^
% subjects increase	51.9	41.0	
Change vs. baseline	−0.2610	−0.5023	
Spearman R			
Spearman *p*-value	0.0542	<0.0001	
Difference in slope (*p*-value)	0.0005 ^$^	<0.0001 ^$^	0.5432 ^&^

^∆^ Wilcoxon *p*-value expressing the significance of the change compared to zero, * *p <* 0.05, ^#^ data are expressed as *p*-values using the Mann–Whitney test, ^$^ value expresses whether slope is different from zero, ^&^ value expresses whether slopes under chitosan and placebo treatments are different.

**Table 11:** Comparison of changes in serum total R_lanosterol induced by chitosan and placebo treatments.

	Placebo ^∆^	Chitosan ^∆^	*p*-Value Chitosan vs. Placebo
All subjects (ng/mg)	0.72 ± 26.59	2.30 ± 29.99	0.4588 ^#^
% subjects decrease	38.2	55.7	0.0654 ^#^
% subjects increase	61.8	44.3	
Change vs. baseline			
Spearman R	−0.1861	−0.4054	
Spearman *p*-value	0.1738	0.0012	
Difference in slope (*p*-value)	0.0004 ^$^	0.0070 ^$^	0.9836 ^&^

^∆^ Wilcoxon *p*-value expressing the significance of the change compared to zero, ^#^ data are expressed as *p*-values using the Mann–Whitney test, ^$^ value expresses whether slope is different from zero, ^&^ value expresses whether slopes under chitosan and placebo treatments are different.

**Table 12:** Comparison of changes in serum total R_desmosterol induced by chitosan and placebo treatments.

	Placebo ^∆^	Chitosan ^∆^	*p*-Value Chitosan vs. Placebo
All subjects (μg/mg)	−0.04 ± 0.25	−0.04 ± 0.16	0.3616 ^#^
% subjects decrease	52.7	62.3	0.3485 ^#^
% subjects increase	47.3	37.7	
Change vs. baseline			
Spearman R	−0.2577	−0.5399	
Spearman *p*-value	0.0575	*p <* 0.0001	
Difference in slope (*p*-value)	<0.0001 ^$^	0.0002 ^$^	0.8998 ^&^

^∆^ Wilcoxon *p*-value expressing the significance of the change compared to zero, ^#^ data are expressed as *p*-values using the Mann–Whitney test, ^$^ value expresses whether slope is different from zero, ^&^ value expresses whether slopes under chitosan and placebo treatments are different.

**Table 13:** Comparison of changes in R_7α-hydroxy-cholesterol induced by chitosan and placebo treatments.

	Placebo ^∆^	Chitosan ^∆^	*p*-Value Chitosan vs. Placebo
All subjects (μg/mg)	0.29 ± 122.85	−28.64 ± 102.15 *	0.5541 ^#^
% subjects decrease	67.3	60.7	0.5622 ^#^
% subjects increase	32.7	39.3	
Change vs. baseline			
Spearman R	−0.3006	−0.6175	
Spearman *p*-value	0.0257	<0.0001	
Difference in slope (*p*-value)	0.1934 ^$^	<0.0001 ^$^	0.2153 ^&^

^∆^ Wilcoxon *p*-value expressing the significance of the change compared to zero, * *p <* 0.05, ^#^ data are expressed as *p*-values using the Mann–Whitney test, ^$^ value expresses whether slope is different from zero, ^&^ value expresses whether slopes under Chitosan and placebo treatments are different.

**Table 14:** Comparison of changes in R_27-hydroxy-cholesterol induced by chitosan and placebo treatment.

	Placebo ^∆^	Chitosan ^∆^	*p*-Value Chitosan vs. Placebo
All subjects (μg/mg)	−7.32 ± 75.67	−12.71 ± 83.28	0.7743 ^#^
% subjects decrease	47.27	57.37	0.3521 ^#^
% subjects increase	52.73	42.63	
Change vs. baseline			
Spearman R	−0.2773	−0.3529	
Spearman *p*-value	0.0404	0.0053	
Difference in slope (*p*-value)	0.0632 ^$^	0.0012 ^$^	0.4827 ^&^

^∆^ Wilcoxon *p*-value expressing the significance of the change compared to zero, ^#^ data are expressed as *p*-values using the Mann–Whitney test, ^$^ value expresses whether slope is different from zero, ^&^ value expresses whether slopes under chitosan and placebo treatments are different.
